# Editorial: Curriculum-based professional learning in early childhood education: conceptualization, implementation and evaluation

**DOI:** 10.3389/fpsyg.2023.1252236

**Published:** 2023-07-25

**Authors:** Weipeng Yang, Alfredo Bautista, Hui Li

**Affiliations:** ^1^Department of Early Childhood Education, The Education University of Hong Kong, Hong Kong, Hong Kong SAR, China; ^2^Shanghai Institute of Early Childhood Education, Shanghai Normal University, Shanghai, China

**Keywords:** curriculum-based professional learning, early childhood education, teacher professional development, conceptualization, implementation, evaluation

## Introduction

The quality of early childhood education (ECE) has a significant impact on children's development and learning outcomes; thus, it is essential to provide effective professional learning opportunities for ECE educators to enhance their knowledge and skills. One promising approach is curriculum-based professional learning (CBPL, Short and Hirsh, 2020), which integrates high-quality curriculum approaches and materials into professional learning activities. This allows teachers to experience the same learning programs as their children and improve their pedagogical practices accordingly. However, there is a lack of empirical evidence on how to implement and evaluate CBPL in ECE settings. To fill this gap, this Research Topic collects a set of empirical explorations of the practices of CBPL in varying contexts and cultures. This editorial synthesizes the findings from this Research Topic, which has addressed the conceptualization, implementation, and evaluation of CBPL in ECE from different perspectives. In particular, this Research Topic comprises three articles that examine the theoretical foundations, frameworks, and components of CBPL; two articles that describe the design and implementation of CBPL initiatives for ECE teachers; and four articles that evaluate the effects of CBPL on teachers and their children. Next, we present brief summaries of each of the studies in the Research Topic according to these categories.

## Theoretical underpinnings of CBPL: framework and components

Some of the studies included in this Research Topic contribute to understanding the key components and processes involved in developing and implementing CBPL initiatives that support ECE principals and teachers in effectively engaging with the curriculum.

First of all, practitioner inquiry and teachers' CBPL are closely related concepts that are both focused on improving teaching practice and student learning outcomes (Campbell and McNamara, 2009). In their paper, Yan and Zhao investigated the relationship between kindergarten school-based practitioner inquiry and preschool teachers' teaching ability, finding that school-based practitioner inquiry positively predicts teaching beliefs and teaching ability. The study highlights the mediating role of constructivist beliefs in teaching, emphasizing the importance of examining the role of teachers' beliefs and teacher inquiry in the development of effective CBPL initiatives.

Tian et al. explored CBPL in China's ECE contexts, with a focus on teachers becoming curriculum designers. This study was drawn upon the Curriculum Design Coherence Model, which is a theoretical framework designed to assist teachers in designing courses that integrate subject concepts, content, and competencies in a coherent way (Rata, 2019; McPhail, 2021). The model's intended usefulness as a curriculum design tool is to contribute to teachers' pedagogical decision-making and professional learning (Rata and McPhail, 2020). By integrating subject concepts, content, and competencies in a coherent way, the model aims to create a curriculum that promotes deep learning and is effective, engaging, and meaningful for learners (McPhail, 2021).

Qian et al.'s study further explored the professional development needs of kindergarten principals in China and found that 70.3% of them had a high need for professional development. The study identifies three profiles of professional development needs and reveals significant rural-urban and public-private differences in the professional backgrounds and professional development needs of the Chinese ECE principals. The findings suggest a noticeable “Matthew effect” in which the poor receive less professional development than the rich. The study's implications for future leader development policy and program development relate to the importance of meeting the professional development needs of kindergarten principals to improve kindergarten quality. This study highlights the need for professional development among educational leaders, who can play a crucial role in supporting and promoting teachers' professional learning within the curriculum.

## Creating and implementing effective CBPL initiatives for ECE teachers

Two other studies focus on developing and evaluating CBPL initiatives for ECE teachers. Saxena and Chiu reported on a CBPL program designed to develop preschool teachers' computational thinking knowledge, attitudes, beliefs, and teaching self-efficacy. They implemented a 6-month training program designed to help teachers develop their understanding and implementation of computational thinking in ECE. The program includes six workshops, a summer training institute, and hands-on practice with related activities including both plugged and unplugged computational thinking. The workshops cover the critical dimensions of computational thinking, pedagogical understanding of computational concepts, practices, and perspectives, and the development of teacher beliefs to support their teaching. Teachers collaborate to design shared curricula and lessons for ECE and develop further learning and teaching resources to institutionalize the integration of computational thinking. Their study highlights the potential of CBPL initiatives to empower ECE teachers to integrate new and innovative approaches into their teaching practice.

In another study, Peng et al. presented the adaptation and validation of a scale for measuring the CBPL community in ECE in China. The Curriculum-Based Professional Learning Community (CBPLC) scale is shown to be a validated tool developed to measure the professional learning community of preschool teachers in the Chinese context. It consists of four factors, including Shared Sense of Purpose, Collective Focus on Children Learning and Development, Collaborative and Reflective Activity, and Deprivatized Practice (Peng et al.). The scale has high reliability and validity, and was found to have a significant positive correlation with teachers' teaching efficacy (Peng et al.). This research contributes to the development of tools and strategies for evaluating and improving the effectiveness of CBPL initiatives in ECE settings.

## Assessing the impact of CBPL on ECE teachers' beliefs and practices and young children's learning and development

The remaining studies explore the impact of CBPL initiatives on ECE teachers and young children's learning and development. First, Yu and Li investigated the transferable skills of children based on STEAM education (practical drawing in this study), providing insights into the potential impact of CBPL initiatives on young children's learning and development. Their research underscores the importance of aligning CBPL initiatives with evidence-based approaches that support children's holistic development.

Zhang et al. explored the relationship between Chinese preschool principal leadership styles and teacher leadership, examining the mediating effect of psychological capital. Their findings highlight the role of leadership in influencing the success of CBPL initiatives and the importance of fostering a supportive and empowering environment for ECE teachers.

Yang and Hong conducted a qualitative exploratory study on early childhood teachers' professional learning about the integration of information and communication technologies in kindergarten curriculum in China. The article discusses the importance of professional learning opportunities for ECE teachers to support their use of digital technologies in the classroom. The study found that ECE teachers in China had received diverse types of professional learning opportunities related to technology use, but there is a need for a teaching-research culture to support their professional learning and to advance current programs. Their research contributes to an understanding of the challenges and opportunities associated with integrating technology into ECE settings and the role of CBPL in supporting this process.

Finally, Xie et al. presented a Chinese model of classroom walkthroughs as an effective strategy for preschool improvement during the COVID-19 lockdowns. Their study emphasizes the potential of CBPL initiatives to support ongoing professional learning and adapt to changing circumstances in the ECE context.

## Conclusion

This Research Topic presents a variety of studies that advance our knowledge of the conceptualization, implementation, and evaluation of CBPL in ECE. Specifically, the papers in this Research Topic emphasize the significance of examining the theoretical foundations of CBPL, designing and implementing effective CBPL initiatives, and measuring their effects on teachers' beliefs and practices as well as children's learning and development. These studies and their findings underscored the potential of CBPL to transform ECE teachers' professional learning experiences and ultimately improve the quality of early learning environments for young children. By building a community of researchers interested in ECE curriculum, pedagogy, and teacher professional development, we can continue to advance our understanding of the opportunities and challenges associated with CBPL and work toward a more effective and equitable ECE system globally.

## Author contributions

WY: conceptualization, writing—original draft preparation, writing—reviewing and editing, and supervision. AB and HL: writing—reviewing and editing. All authors contributed to the article and approved the submitted version.
